# Clinicopathological features and prognosis of gastroenteropancreatic neuroendocrine neoplasms in a Chinese population: a large, retrospective single-centre study

**DOI:** 10.1186/s12902-017-0190-6

**Published:** 2017-07-13

**Authors:** Meng Zhang, Ping Zhao, Xiaodan Shi, Ahong Zhao, Lianfeng Zhang, Lin Zhou

**Affiliations:** 1 Department of Gastroenterology, the First Affiliated Hospital of Zhengzhou University, No.1, East Jianshe Road, Zhengzhou, 450052 China; 2 Department of Pathology, the First Affiliated Hospital of Zhengzhou University, No.1, East Jianshe Road, Zhengzhou, 450052 China

**Keywords:** Neuroendocrine neoplasms, Neuroendocrine tumors, Gastro enteropancreatic neuroendocrine tumors, Neuroendocrine cancers, Carcinoid tumor, Gastrinoma, Islet cell tumor

## Abstract

**Background:**

Gastroenteropancreatic neuroendocrine neoplasms (GEP-NENs) are the most common type of neuroendocrine tumors, accounting for more than half of neuroendocrine neoplasms (NENs). We performed a retrospective study in our center to investigate the clinicopathological features, risk factors of metastasis, and prognosis of GEP-NENs in a Chinese population.

**Methods:**

Four hundred forty patients with GEP-NENs treated at the First Affiliated Hospital of Zhengzhou University between January 2011 and March 2016 were analyzed retrospectively. Multivariate logistic regression was performed to identify independent risk factors for metastasis of the tumors. The Kaplan-Meier method was used for survival analysis, and log-rank tests for comparisons among groups.

**Results:**

Primary sites were the stomach (24.3%), rectum (24.1%), pancreas (20.5%), esophagus (12.3%), unknown primary origin (UPO-NEN) (8.0%), duodenum (6.1%). Three hundred eighty-nine of the 440 GEP-NENs cases (88.4%) were non-functional tumors, and patients had non-specific symptoms, which could have led to delay in diagnosis and treatment. Neuroendocrine tumor, neuroendocrine carcinoma, and mixed adenoendocrine carcinoma were 56.8%, 33.2% and 3.2%, respectively, of the cases. One hundred thirty (29.5%) of the tumors were G1, 120 (27.3%) G2, and 190 (43.2%) G3. The immunohistochemical positive rate of synaptophysin was 97.7% and of chromogranin 48.7%. Logistic regression analysis revealed that the diameter and pathological classification of tumors were the most important predictors for metastasis. The median survival time was 34 months for patients with well-differentiated neuroendocrine tumors grade G3 and 11 months for poorly differentiated neuroendocrine carcinoma. The median survival time of patients with localized disease, regional disease, and distant disease was 36 months, 15 month, and 6 months, respectively.

**Conclusions:**

This study constitutes a comprehensive analysis of the clinicopathological features of GEP-NENs in a Chinese population. GEP-NENs may occur at any part of the digestive system. The diameter and pathological classification of tumor are the most important predictors for metastasis. The prognosis is poor for patients with poorly differentiated neuroendocrine cancers and distant metastases.

## Background

Neuroendocrine neoplasms (NENs), which originate from neuroendocrine cells, comprise a heterogeneous family with a broad spectrum of clinical behavior [1]. The neoplasms occur in diverse sites throughout the body, and more than half are gastroenteropancreatic NENs (GEP-NENs) [2]. According to the Surveillance, Epidemiology, End Results database (SEER), which has the largest epidemiologic series, the incidence of NENs has risen substantially over the past 30 years [2]. Although the prevalence of GEP-NENs seems to be increasing in China, there is no accurate database of the characteristics of GEP- NENs in Chinese patients.

In 2010, the World Health Organization [3] proposed a new classification of NENs, with comparisons of clinical, pathological, therapeutic, and prognostic factors. In western countries, the epidemiology, treatment, and survival rates of NENs have been well-studied [1, 2], but comparable information in Asian populations is limited [4, 5]. In order to investigate the clinical pathological characteristics, risk factors of metastasis, and prognosis of NENs in a Chinese population, we performed a comprehensive retrospective review of the recent 5-year experience with this disease in our center.

## Methods

### Patients

The study was conducted on 440 patients from the First Affiliated Hospital of Zhengzhou University between January 2011 and March 2016. The study was approved by the hospital’s Ethics Committee, and informed consent was obtained from all patients. All patients had received a pathological diagnosis of GEP-NENs according to the World Health Organization classification [3] and the China Consensus Guideline [6]. Collected information consisted of clinical characteristics (gender, age, location of tumors, and symptoms); diagnostic procedures (endoscopic and radiographic); tumor characteristics (size, grading, histopathology of primary tumor, metastases); treatments; and follow-up.

The pathological diagnosis of the NENs depended on typical morphological findings and immunohistochemical staining of chromogranin (CgA) and/or synaptophysin (Syn) [7]. Grading was based on morphological criteria and tumor proliferative activity. According to the Ki-67 index, the grading was G1, G2 and G3 ≤ 2%, 3 ~ 20%, >20%, respectively. Similarly, tumors with mitotic rates of less than two in 10 high-power fields (HPF) were classified as G1, 2 ~ 20/HPF as G2, and >20/HPF as G3. If the grading of the Ki-67 index differed from that of the mitotic rate, the higher of the two was given priority. Therefore, GEP-NENs were classified as neuroendocrine tumor (NET) (G1 and G2), neuroendocrine carcinoma (NEC) (G3), and mixed adenoendocrine carcinoma (MANEC) (G3) [3, 4]. The well-differentiated G3 NENs (Ki-67 positive index >20%; generally less than 60%) were classified as well-differentiated NET (NET G3) [8, 9].

### Statistical analysis

All statistical analyses were performed using SPSS 17.0 for Windows (IBM Corporation. Armonk, NY, USA). Normally distributed continuous variables were expressed as mean and standard deviation, and statistical differences between groups were assessed with the independent samples t-tests. Differences in categorical variables were compared with chi-square test or Fisher ‘s exact test. Multivariate logistic regression was performed to identify independent risk factors for tumor metastasis. Overall survival was defined as the time from diagnosis to death or, in living patients, the time to last follow-up. Survival curves were drawn according to the Kaplan-Meier method, and differences between subgroups were assessed with the log-rank test. *P*-values<0.05 were considered statistically significant.

## Results

### Clinical features

Among the 440 patients with GEP-NENs, 259 (58.9%) were men and 181 (41.1%) were women; the male to female ratio was 1.43. Ages ranged from 9 to 86 years, and the mean age was (54.3 ± 13.5) years. The mean age of men was (55.5 ± 13.5), women was (52.7 ± 13.3). The most common tumor site was the stomach (24.3%, 107/440), followed by the rectum (24.1%, 106/440), pancreas (20.5%, 90/440), esophagus (12.3%, 54/440), unknown primary origin (UPO-NEN) (8.0%, 35/440), duodenum (6.1%, 27/440), and other sites: appendix, jejunum/ileum, and colon (4.7%, 21/440).

Non-functional tumors comprised the majority of GEP-NENs (389/440, 88.4%); the other 51 (11.6%) were functional. The most frequent initial presentation was abdominal pain (101/440, 23%), which was not specific for the diagnosis of tumor, followed by dysphagia (45/440, 10.2%), bleeding (38/440, 8.6%), diarrhea (19/440, 4.3%), jaundice (16/440, 3.6%), and abdominal distention. Forty-one (9.3%) cases were found during routine physical examination. Insulinoma comprised 90.2% (46/51) of functional tumors, all of which were located in the pancreas, and typical symptoms were those of hypoglycemia and epileptic seizure. Seven patients with insulinoma were treated as epilepsy before the diagnosis of NENs, and 2 cases were initially treated as psychiatric disorders. The other functional tumors were gastrinoma (3/51, 5.9%) and glucagonoma (2/51, 3.9%), expressed as multiple refractory peptic ulcer, diarrhea, secondary diabetes mellitus and cutaneous erythema. There was no patient presented with carcinoid syndrome in our study.

### Imaging studies

The results of imaging examinations are summarized in Table 1. All imaging examinations can be found in any grade of tumors. Endoscopy, endoscopic ultrasound (EUS) and positron emission computed tomography imaging (PET-CT, using with 18F-FDG) were positive in >90% of cases. Magnetic resonance imaging was the least often positive (79.5%). But MRI and PET-CT, was performed in only about 10% of patients, respectively. MRI is mainly used for the detection of pancreatic and liver tumors and PET-CT for tumors in any part of the digestive system. EUS was performed on 41 patients, of which a lesion was found in 38 patients. At endoscopy, which is used for the detection of gastrointestinal tract tumors, the GEP-NENs usually appeared as ulcers or polypoid prominences. Ultrasound and endoscopic ultrasound (EUS) usually revealed the GEP-NENs as hypoechoic and well-vascularized masses. By computed tomography (CT) and magnatic resonance imaging (MRI), the tumors appeared as local space-occupying lesions, with heterogeneous enhancement on contrast-enhanced CT. PET-CT usually revealed high glucose metabolism in GEP-NENs, especially in poorly differentiated NENs. Seven tumors, initially identified in the liver, were found located in the pancreas by EUS-guided fine-needle biopsy.

**Table 1:** Characteristics of imaging studies

Imaging study	Site	Cases tested (n)	Positive tests	
			n	%
Endoscopy	gastrointestinal	226	224	99.1%
EUS	pancreas, duodenum, stomach	41	38	92.7%
Ultrasound	pancreas, liver, biliary tree	165	143	86.7%
CT scan	all of above	321	274	85.4%
MRI	pancreas, liver	39	31	79.5%
FDG PET-CT	all of above	29	27	93.1%

*EUS* endoscopic ultrasonography, *CT* computed tomography, *MRI* magnetic resonance imaging, *FDG PET-CT* fluorodeoxyglucose positron emission computed tomography imaging

### Histopathologic characteristics

The histopathologic characteristics (size, World Health Organization 2010 classification, and metastases) of the 440 GEP-NENs are given in Table 2. The most common tumor type was NET (250, 56.8%), followed by NEC (146, 33.2%) and MANEC (14, 3.2%); the other 30 cases of G3 were classified as NET G3. Local infiltration and lymphatic metastasis occurred in 63% (277/440) and 12.3% (54/440) of patients, respectively. Distant metastases were found in 90 (20.5%) patients at initial diagnosis; during follow-up, the number increased to 109 (24.8%). Distant metastases were present at initial diagnosis in 38.4% (73/190) of patients with G3 tumors. The most frequent site of distant metastasis was liver (67/109, 61.5%), followed by peritoneum (18.3%, 20/109), lung (10.1%, 11/109) and bone (6.4%, 7/109). Among the 67 patients with liver metastases, 55 presented with synchronous lesions and 12 with metachronous lesions during follow-up. The positive rates of immunohistochemistry of Syn and CgA were 97.7% (416/426) and 48.7% (135/277, respectively.

**Table 2:** Histopathological characteristics of the GEP-NENs (*n* = 440)

	Stomach	Esophagus	Duodenum	Jejunum/ileum	Colon	Appendix	Rectum	UPO-NEN	Pancreas	Total
	*n* = 107	*n* = 54	*n* = 27	*n* = 8	*n* = 5	*n* = 8	*n* = 106	*n* = 35	*n* = 90	*n* = 440(%)
Size										
<2 cm	24	5	15	1	0	3	67	4	37	156(35.5)
2 ~ 4 cm	12	10	3	2	0	0	2	6	16	51(11.6)
>4 cm	31	12	3	3	2	1	1	8	11	72(16.4)
Unclear	40	27	6	2	3	4	36	17	26	161(36.6)
WHO2010										
G1	17	0	6	1	1	5	70	3	27	130(29.5)
G2	21	1	11	2	0	2	26	7	50	120(27.3)
G3	69	53	10	5	4	1	10	25	13	190(43.2)
Metastases										
Local	43	16	18	4	2	8	94	19	73	277(63)
Loco-regional	28	14	1	2	1	0	3	4	1	54(12.3)
Distant	36	24	8	2	2	0	9	12	16	109(24.8)

The clinicopathologic characteristics related to metastasis were listed in Table 3. The risk factors of GEP-NENs metastases were then analyzed by the logistic regression method. Multivariate logistic regression analysis revealed that the diameter and pathological classification of tumors were the most important predictors for metastasis (Table 4).

**Table 3:** Clinicopathological characteristics related to metastasis

Pathologic characteristics	Total	Metastasis	Non-metastasis	*χ* ^*2*^	*P*
Sex					
Male	259	112(43.2%)	147(56.8%)	10.370	<0.01
Female	181	51(28.2%)	130(71.8%)		
Age					
≤ 50	158	33 (20.9%)	125 (79.1%)	27.602	<0.01
>50	282	130 (46.1%)	152 (53.9%)		
Site					
Stomach	107	64(59.8%)	43(40.2%)	67.249	<0.01
Rectum	106	12(11.3%)	94(88.7%)		
Pancreas	90	17(18.9%)	73(81.1%)		
Functional status					
Functional	51	2 (3.9%)	49 (96.1%)	25.556	<0.01
Nonfunctional	389	161 (41.4%)	228 (58.6%)		
Tumor diameter					
≤ 2 cm	156	14(9.0%)	142(91.0%)	80.879	<0.01
2-4 cm	51	23(45.1%)	28(54.9%)		
≥ 4 cm	72	47(65.3%)	25(34.7%)		
Tumor grading					
G1	130	4(3.1%)	126(96.9%)	182.475	<0.01
G2	120	22(18.3%)	98(81.7%)		
G3	190	137(72.1%)	53(27.9%)		
Tumor type					
NET	250	26(10.4%)	224(89.6%)	182.746	<0.01
NEC	146	113(77.4%)	33(22.6%)		
MANEC	14	8 (57.1%)	6 (42.9%)

**Table 4:** The logistic regression for the relationship between the clinicopathological characteristics and metastasis

Variable	Coefficient	Standard Error	Wald *χ* ^*2*^	*P*	Odds ratio	95%CI	
Tumor diameter	0.790	0.239	10.909	0.001	2.203	1.379	3.521
Grading	1.998	0.326	37.510	<0.01	7.373	3.890	13.974
Constant	−6.887	0.811	72.106	<0.01	0.001	--	--

### Therapeutic interventions

About two-thirds of the patients (62.5%; 275/440) underwent an operation with curative intent or palliation; 50 patients were treated with endoscopic radical surgery, mainly for rectal lesions. Seventy-three patients received chemotherapy, 34 of whom received postoperative adjuvant chemotherapy. The combination of cisplatin and etoposide was the most widely used chemotherapeutic agents. Six patients received octreotide, a somatostatin analogue, as a biological therapy combined with surgery or chemotherapy. Local-regional therapies, such as transcatheter hepatic arterial chemoembolization, radiofrequency, or other ablative techniques were used in eight patients.

### Survival and prognostic factors

**Fig. 1:**
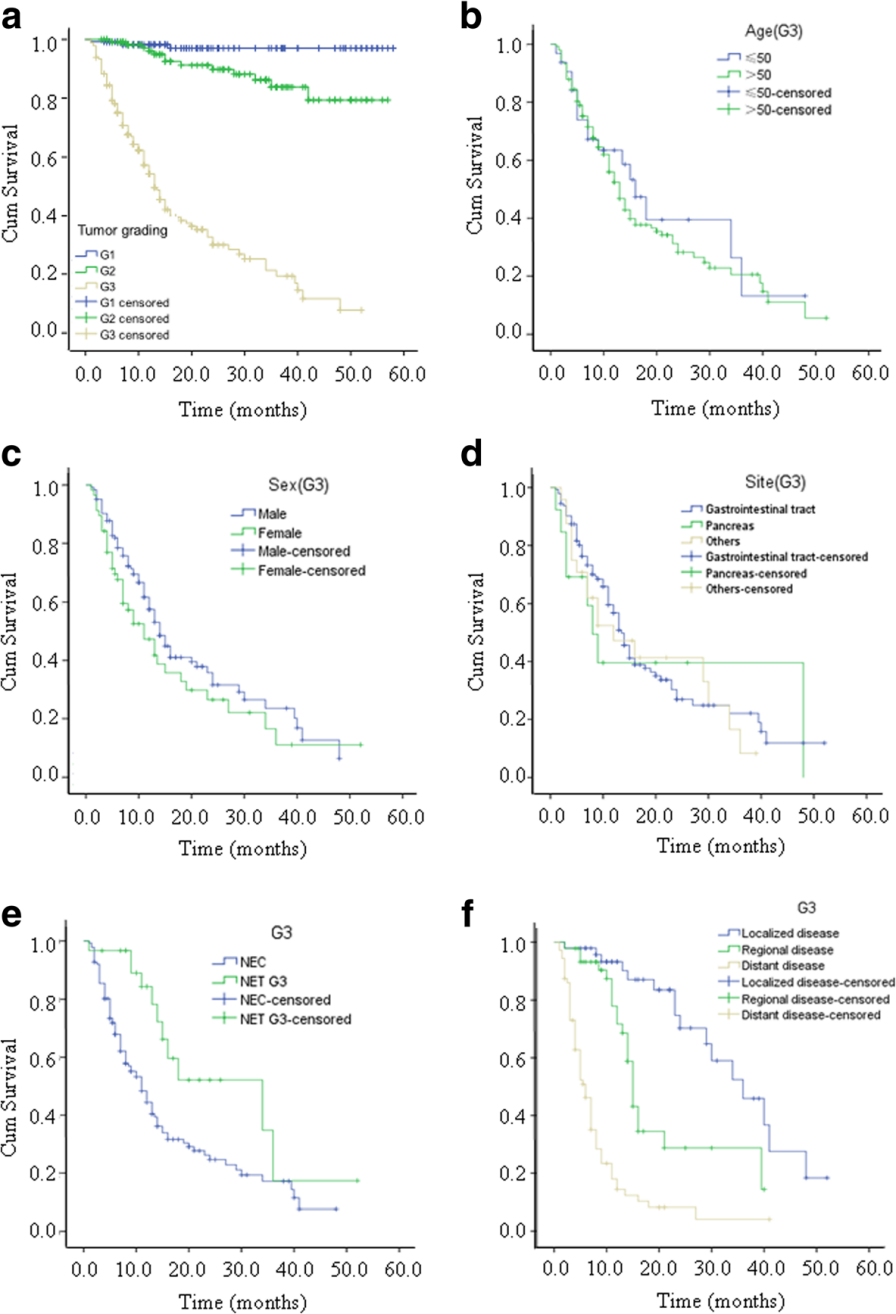
Kaplan-Meier analysis of overall survival. **a** Overall survival by histological grading. **b** Overall survival by age of of patients with G3 tumors. **c** Overall survival by sex of patient with G3 tumors. **d** Overall survival by site of G3 tumors. **e** Overall survival by differentiation of G3 tumors. **f** Overall survival by characteristics of metastasis of G3 tumors

Four hundred fourteen of the 440 patients were followed for periods of 3–60 months. Due to the short follow-up time and a low number of deaths in NET (G1 and G2) patients, median survival time was not obtained for them during the observation period. The 1-, 3- and 5- year survival rates of all patients were 78.7%, 60.8% and 54.5, respectively. The 1-, 3- and 5- year survival rates of patients with G3 lesions were 54.3%, 19.4% and 7.8%, respectively. The major causes of death were tumor-related complications (82.7%), treatment-related adverse events (13.4%), and other diseases (3.9%). For patients with G3 NENs, age, gender, primary tumor site, differentiation, and characterization of metastasis were analyzed in order to identify prognostic factors for survival (Table 5). Univariate analysis confirmed that patients with NET G3 and patients without regional or distant metastasis survived longer than did other patients with NENs G3, but age, gender, and primary tumor site had no discernable impact on overall survival. Median overall survival among all the patients with G3 NENs was 13.0 months, and survival was significantly longer for these patients (median 34 months) than for those with NEC (median 11 months). Median overall survival of patients with localized G3 NENs was 36 months, 15 months for patients with regional disease, and 6 months for patients with distant disease. Survival curves are displayed in Fig. 1, a-f.

**Table 5:** Overall survivals of G3-NENs

Factors	Overall survival				
	Number	Mean(months)	95%CI	*χ* ^*2*^	*P*
All patients	180	13	10.9-15.1		
Sex				2.386	0.122
Male	123	14	11.7-16.3		
Female	57	11	6.4-15.6		
Age				0.466	0.495
≤ 50	32	16	11.2-20.8		
>50	148	13	11.0-15.0		
Site				0.520	0.771
Gastrointestinal tract	143	13.5	11.8-15.2		
Pancreas	13	8	5.0-11.0		
Others	24	12	2.9-21.1		
Differentiation				9.186	0.002
NEC	137	11	8.5-13.5		
NET G3	30	34	10.7-57.3		
Metastasis				85.305	0.000
Local	48	36	25.9-46.1		
Loco-reginal	45	15	13.9-16.2		
Distant	71	6	4.7-7.3

## Discussion

This retrospective study explored the clinicopathological characteristics, risk factors of metastasis, treatment, and prognosis of GEP-NENs in a relatively large population of Chinese patients. Tumors located at any possible site within the digestive system were analyzed. In addition, the NENs G3 were classified as NET G3 (well-differentiated NET with a G3 grading) or NEC, and the prognoses of these were compared. The study will contribute to establishing a database of the epidemiology, clinical pathological features, treatment, and prognosis of GEP-NENs in Chinese or Asian patients. It also will permit comparisons between GEP-NENS in these populations and those in western nations.

As others have reported [2], we found that most NENs are present in the gastrointestinal tract. The distribution of GEP-NENs in our population (stomach > rectum > pancreas > duodenum) was similar to that found in Korean [10], Japanese [11], and other Chinese populations [4, 12, 13]. A large-scale analysis of GEP-NENs in the SEER database from the United States in North America found that the rectum and jejunum/ileum were the most common sites for NENs [2]. Ethnic, regional and sample-size differences may lead to differences in the reported distribution of the primary sites of GEP-NENs.

NENs can be classified as functional and non-functional tumors according to the presence or absence of symptoms associated with hormone overproduction [14]. In this study, the majority of tumors were non-functional, which may have led to misdiagnosis or delay in making the diagnosis and treating the patients promptly. The study found also that insulinoma comprised the largest number of functional NENs and accounted for about one-half of all NENs. None of the patients presented with carcinoid syndrome due to overproduction of 5-hydroxytryptamine, a finding similar to a low reported frequency of these tumors in Asian populations [4, 12, 13]. However, the incidence of carcinoid syndrome (11.5% to 31.1%) in the Western population is relatively high [15–17]. Carcinoid syndrome occurs mainly in the neuroendocrine neoplasms of jejunum/ileum. In the western population, the proportion of neuroendocrine neoplasms of jejunum/ileum is high, which is only 1.8% in our study. These data also indicated that functional NENs were mainly located in the pancreas, and the gastrointestinal neuroendocrine neoplasms were mainly non-functional. Several studies have used serum CgA as a circulating biomarker of GEP-NENs, with sensitivity and specificity rates in the range of 60% to 100% [18]. Serum CgA was mot measured in our series, but we favor measuring it since it is a simple screening test and can shorten the time needed to make the diagnosis of a NEN.

Since non-functional GEP-NENs in the early stage often have no specific symptoms, imaging examinations are especially important in locating the tumors and assessing their extent. CT scan was the most frequently used imaging modality, whereas endoscopy had the highest yield of tumor detection (99.1%). Because of its convenience and non-invasive nature, ultrasound was chosen as the first screening method for solid organs, where the detection rate was 87%. EUS provides unique advantages in evaluating the upper gastrointestinal tract and pancreas, especially for tumors less than 1.0 cm in diameter and micrometastases [19]. The 92.3% detection rate of GEP-NENs in our series is within the range (91.9% to 97.4%) reported by others [4, 13]. The most common primary site of metastatic liver NENs is the pancreas, so EUS-guided fine-needle biopsy of the pancreas in patients with metastatic liver NENs is helpful for early detection of the primary lesion. Somatostatin receptor scintigraphy is considered a comprehensive imaging modality for many neuroendocrine tumors [20, 21], but, unfortunately, this method is not available in our institution.

The definitive diagnosis of GEP-NENs depends on the pathological analysis of biopsy, including cell morphology and immunohistochemical staining. The World Health Organization revised its nomenclature and classification of GEP-NENs in 2010 [3], and China reported its own classification system soon thereafter [6]. Rates of positive immunohistochemical staining for Syn (97.7%) and CgA (48.7%) in our series indicate that Syn has high sensitivity, and CgA has high specificity [22].

In our series, the rate of distant metastases (20.5% initially and 24.8% during follow-up) was modestly lower than the rate reported from Spain (44%) [23] but in the range reported from the United States (21%) [2] and in other Chinese series (10.4% to 23.0%) [4, 12, 13]. The liver was the most frequent site of metastatic tumors. The rate of distant metastases at diagnosis was high, which indicates that GEP-NENs, especially the non-functional tumors, were occult, a characteristic that could have led to delayed diagnosis and increased risk of metastasis. The rate of transfer factors of GEP-NENs was related to location of the primary tumors, with the metastasis rate of gastric NENs significantly higher than that of pancreatic or rectal tumors. The reported rate of metastasis of pancreatic NENs (69.2%) in a Western population [24] was higher than in our series (18.9%); the possible reasons for this difference could be differences in the ratio of non-functional to functional pancreatic NENS, with differences in time duration between onset of symptoms and diagnosis, or sample size. Also, we admit that the rate of missed diagnosis of pancreatic NENs in China may be high.

Surgical treatment is the first choice for GEP-NENs, even if there are nodal or distant metastases. When possible, the primary tumor should be removed, lymph nodes dissected, and distant metastases excised [25]. In this study, 275 patients underwent surgical treatment, including radical surgery and palliative surgery, with 50 patients treated by endoscopy. Early diagnosis is crucial in order that resection can be performed before local invasion or distant disease occurs.

Chemotherapy is the first treatment option for poorly differentiated or rapidly progressive, advanced GEP-NENs. In our series, as in other reports [26], the combination of cisplatin and etoposide was the most widely used chemotherapy regimen. Radiofrequency ablation, transcatheter hepatic arterial chemoembolization, or other ablative treatments, which can be used to treat liver lesions, were used in only 8 patients in our series, and only 6 patients were treated with biological therapy. Limited financial resources in our area may have contributed to the infrequent use of newer or experimental therapies for GEP-NENs such as peptide receptor radionuclide therapy and targeted agents.

The prognosis of GEP-NENs is more favorable than that of adenocarcinomas of the digestive system. In our series, the overall 5-year survival rate was 54.5%, which is similar to that quoted the SEER registry and in Chinese data [2, 4], but lower than the rate in some Europe countries [23]. These differences may be due to the ethnic, regional, or simple-size differences. Due to the short follow-up time and a low number of deaths in NET (G1 and G2) patients, we did not determine median survival times during the observation period, and limited our survival estimates to patients with NENs G3; their 5-year survival rate was 7.8%, which is similar to rates of 6%–11% in European series [27]. Very few data comparing NET G3 and NEC are available [8, 9]. In our series, there was a significant difference in the survival time between the G3 NET and NEC (34 months vs 11 months), which are similar results to those of other series [9, 28, 29]. While most NENs G3 are poorly differentiated, a subgroup of well-differentiated NET with G3 grading is not reflected in the latest Word Health Organization classification. It has been suggested that these two high-grade cancers differ in prognosis, somatostatin receptor scintigraphy uptake and response to chemotherapy regimens [29, 30] and therefore should be classified separately. The small size of our series of NET G3 tumors precluded doing a multivariate analysis to estimate their independent prognostic factors; evaluation in a larger population of such tumors is needed. Unsurprisingly, we found that distant metastasis of GEP-NENs was associated with a poor outcome; thus, early diagnosis of the tumors is very important in improving patients’ prognosis.

## Conclusions

The results of this study provide a comprehensive analysis of the clinicopathological features of GEP-NENs in a Chinese population. It was found that GEP-NENs may originate from any part of the digestive system, and the majority are non-functional tumors, whose early symptoms are occult, thus often resulting in delay in the diagnosis being made. Tumor diameter and classification are important factors in predicting metastasis. The prognosis of GEP-NENs is more favorable than that of gastrointestinal carcinomas, but the prognosis is poor for patients with high-grade poorly differentiated NEC and distant metastases. It is our hope that this extensive analysis of GEP-NENs will improve physicians’ knowledge of the tumors and result in earlier recognition and treatment for Chinese patients. And limited financial resources in our area may lead to the infrequent use of newer or experimental therapies for GEP-NENs. Perhaps this could be a probable explanation on poorer prognosis compared to Western data.

## Abbreviations

CgA: Chromogranin
CT: Computed tomography
EUS: endoscopic ultrasound
GEP-NENs: Gastroenteropancreatic neuroendocrine neoplasms
HPF: High-power fields
MANEC: Mixed adenoendocrine carcinoma
MRI: Magnatic resonance imaging
NEC: Neuroendocrine carcinoma
NENs: Neuroendocrine neoplasms
NET G3: Well-differentiated NET with a G3 grading
NET: Neuroendocrine tumor
PET-CT: positron emission computed tomography imaging
SEER: Surveillance, Epidemiology, End Results database
Syn: Synaptophysin
WHO: World Health Organization
